# Analysis of patient health questionnaire-9 (PHQ-9) based depression prevalence according to a discordance between quantitative urinary cotinine levels and self-report of second-hand smoke exposure among adults: A cross-sectional study

**DOI:** 10.1016/j.heliyon.2024.e32125

**Published:** 2024-05-29

**Authors:** Hyun-Seung Lee, Young-Jin Lee, Ji-Hyun Cho, Do-Sim Park

**Affiliations:** a Department of Laboratory Medicine and Institute of Wonkwang Medical Science, School of Medicine, Wonkwang University, Iksan, South Korea; b Wonkwang Institute of Clinical Medicine, Wonkwang University Hospital, Iksan, South Korea

**Keywords:** Cotinine, Self-report, Second-hand smoke, Depression, PHQ-9, Stress

## Abstract

**Background:**

Second-hand smoke (SHS) exposure appears to be more common among individuals with depression. However, self-report of SHS exposure is an inaccurate classification compared to confirming SHS exposure using urinary cotinine (UC). Additionally, the dose-response relationship between depression and UC is controversial.

**Methods:**

The severe stress rate and depression prevalence was estimated among 14530 Korean participants aged ≥19 years using data patient health questionnaire-9 (PHQ-9) and on UC from the Korean National Health and Nutrition Examination Survey. Measured UCs were divided into four categories: UC– (≤0.3 μg/L), UC± (0.4 μg/L–0.9 μg/L), UC+ (1.0 μg/L–11.9 μg/L), and UC++ (≥12.0 μg/L).

**Results:**

About 55.0 % participants were female and participants’ mean age was 51.1 years. Non-smokers were 80.3 %. Among non-smokers, non-SHS exposure participants (SR–) and SHS exposure participants (SR+) were 83.0 % and 17.0 %, respectively. When UC– was used as the reference subgroup, the UC++ subgroup showed a higher depression prevalence, whereas the UC ± subgroup showed a lower prevalence. In the same UC categories, the depression prevalence and severe stress rate were higher among females than among males. Furthermore, the SR + subgroup had a higher severe stress rate than the SR– subgroup.

**Conclusions:**

Our study showed a paradoxical reduction in the depression prevalence and severe stress rate in the UC ± subgroup compared to the UC– subgroup. Additionally, the dose-response relationship between the SHS exposure biomarker and the depression prevalence was not linear. Our study indicates that an emotional stress-based model may be more appropriate for explaining the relationship between depression and SHS exposure.

## 1 Introduction

Tobacco smoking is a well-known risk factor for physical illnesses such as asthma, respiratory tract infections, and various cancers [[1], [2], [3]]. Additionally, tobacco smoking is more common among individuals with mental health problems, particularly depression [4]. A previous study found that 31 % of depression prevalence were smokers compared to 12.5 % of smokers in the general U.S. population [5]. Smokers with depression are less likely to maintain smoking cessation and have higher chances of relapse [6]. One hypothesis explaining the relationship between smoking and depression is that chronic exposure to tobacco smoke among smokers may lower their dopamine and gamma-aminobutyric acid (GABA) levels, which are associated with depression [7,8]. Another hypothesis suggests that depression leads to tobacco smoking because nicotine dependence may have mood-regulating effects [9]. Additionally, some environmental or genetic risk factors may be common among smokers and patients with depression [10,11].

Second-hand smoke (SHS) exposure, which is defined as the combination of smoke from the burning end of a cigarette and the smoke exhaled by smokers [12], also appears to be related with mental health problems. Several studies [[13], [14], [15], [16], [17], [18]] indicate that self-reported SHS exposure is related to depression or depressive symptoms. The classification of self-reported SHS exposure is an inaccurate method compared to confirming SHS exposure using tobacco-specific biomarkers [19] because it is subjective and has a low correlation with quantitative values of serum cotinine or urinary cotinine concentration (UC) [20]. However, emotional experience of avoidable or distressing exposure may be a better predictor of mental health effects than objective UC. Therefore, it is essential to identify the factor that has higher association with depression or depressive symptoms between self-reported SHS exposure and objectively measured UC.

A few studies have reported that SHS exposure confirmed by blood cotinine is related with depressive symptoms among non-smokers, even after adjusting for gender [21,22]. Conversely, a report from two studies conducted in the Netherlands indicates that plasma cotinine levels are not related with either depression or depressive symptoms [23]. Several studies have shown a correlation between UC confirmed SHS exposure and depressive symptoms only among females [24,25]. Therefore, the dose-response relationship between depression and objectively assessed UC, which indicates biological SHS exposure, remains controversial [15].

Various hypotheses have been proposed to explain the relationship between depression and SHS exposure. Some studies have evaluated the association between severe stress and SHS exposure [22,[26], [27], [28]]. A meta-analysis of 11 cross-sectional studies revealed that SHS exposure is significantly associated with depressive symptoms and psychological distress [15]. Similar to first-hand smoke exposure, SHS exposure may also be related with lower dopamine and GABA levels [29]. The relationship between SHS exposure and inflammatory factors has been examined [30] because depression is related with a chronic low-grade inflammatory response [31]. However, the mechanism underlying the association between depression and SHS exposure remains unclear.

The current study estimated the severe stress rate and depression prevalence among Korean adults using the patient health questionnaire-9 (PHQ-9) [32] data obtained from the Korean National Health and Nutrition Examination Survey (KNHANES). We then compared the depression prevalence or severe stress rate in each subgroup according to the self-report of SHS exposure, categorized UC, and gender. Therefore, we aimed to elucidate the dose-response relationship between categorized UC and depression or severe stress and explain the mechanism underlying the relationship between depression and SHS exposure.

## 2 Methods

### 2.1 Study participants

**Fig. 1:**
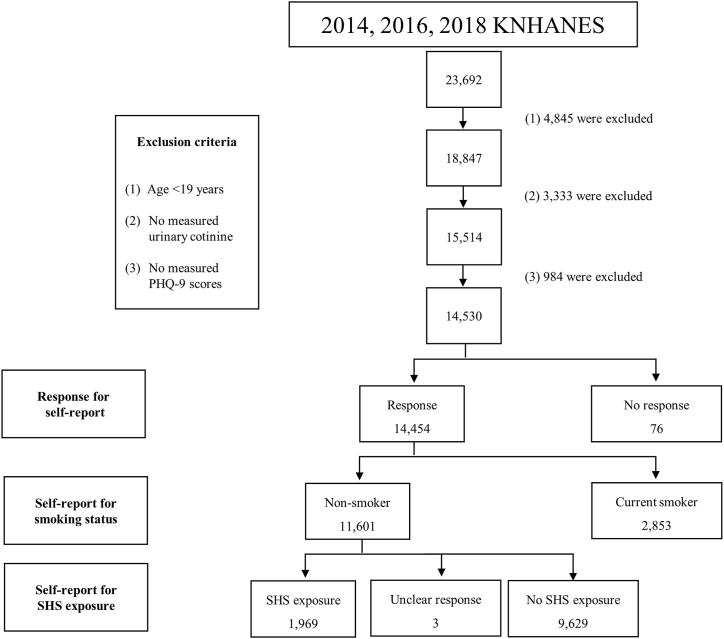
Schematic flowchart of the study design and exclusion criteria.

Fig. 1 illustrates a schematic flowchart of the study design and exclusion criteria. Of the total 23692 individuals who enrolled in the 2014, 2016, and 2018 KNHANES surveys, 4845 participants aged under 19 years were excluded (Supplementary Table S1). Of the 18847 adult participants, we excluded 3333 and 984 participants because of missing UC and PHQ-9 data, respectively. Finally, the current study enrolled 14530 participants aged ≥19 years with measured UCs and PHQ-9 scores.

### 2.2 Self-report of SHS exposure

In the current study, the classification of self-reported SHS exposure was the same as in a previous study [20]. Briefly, SHS exposure participants (SR+) were defined as non-smoker participants who reported a history of SHS exposure at the workplace or home. Non-SHS exposure participants (SR–) were defined as non-smoker participants who clearly reported no history of SHS exposure at the workplace and home. Non-smoker participants with unclear responses to SHS exposure were defined as non-smoker participants who are neither SR + nor SR–.

### 2.3 Urinary cotinine analysis

The measurement methods of UC and their diagnostic performance were the same as in a previous study [20]. Briefly, the measurement of UC was performed using gas chromatography-tandem mass spectrometry (GC-MS/MS) in 2014. In 2016 and 2018, a high-performance liquid chromatography-tandem mass spectrometry (HPLC-MS/MS) system was used. The limit of detection (LoD) of both systems was same as 0.27 μg/L.

### 2.4 Categorization of urinary cotinine concentrations

Measured UCs were divided into the following four categories: UC– (≤0.3 μg/L), UC± (0.4 μg/L–0.9 μg/L), UC+ (1.0 μg/L–11.9 μg/L), and UC++ (≥12.0 μg/L). The categorization values were chosen because UC of 0.3 μg/L is close to the LoD and UC of 1.0 μg/L is close to the value of 75 % of non-smokers without SHS exposure in the current study. UC of 12.0 μg/L was close to the optimal cut-off value in the 2018 KNHANES data to distinguish current smokers from non-smokers [20]. Therefore, UC–, UC±, UC+, and UC++ were considered as biologically indicators of no SHS exposure, minimal-dose SHS exposure, mid-dose SHS exposure, and high-dose SHS exposure similar to the current smoker level, respectively.

### 2.5 The depression prevalence using the PHQ-9 score

In the current study, participants with depression was defined as those with the PHQ-9 score ≥10 (Supplementary Table S2). The depression prevalence was estimated as the proportion of the sample that meet the criteria for depression. in each subgroup.

### 2.6 The severe stress rate

In the current study, participants with severe stress were defined as those who reported experiencing severe stress or very severe stress in their daily lives (Supplementary Table S2). The severe stress rate was estimated as the percentage of participants with severe stress out of the total number of participants in each subgroup.

### 2.7 Statistical analysis

Due to methodological differences in the LoD in the current study, each value ≤ 0.3 μg/L was converted to 0.3 μg/L. Categorical data was analyzed using Fisher's exact test or the chi-square test with Bonferroni-adjusted post-hoc test. Binominal logistic regression analysis was used to calculate the odds ratio (OR) for the depression prevalence or severe stress rate with age, gender, education level, household income, type of house, and type of marriage, SR, categorical UC, and quantitative UC, as covariates (Supplementary Table S3). The GraphPad Prism (version 9.4.1.; GraphPad Software, La Jolla, CA, USA) and Statistical Package for the Social Sciences version 25.0 (IBM Corporation, Armonk, NY, USA) were used for graphs and statistical analyses. A *p* value of <0.05 was considered as statistically significant.

## 3 Results

### 3.1 The characteristics of study participants

Table 1 displays the characteristics of study participants. Of the 14530 enrolled participants, females were 55.0 % (7995/14530), and the mean age with standard deviation (SD) was 51.1 ± 16.5 years. Of the 14454 participants who self-reported their smoking status, 80.3 % (11601/14454) were non-smokers. Among non-smokers, SR– and SR+ were 83.0 % (9629/11601) and 17.0 % (1969/11601), respectively. Three participants reported unclear responses to the SHS exposure. Among 2853 current smokers, 87.1 % (2484/2853) were daily smokers and 12.9 % (369/2853) were non-daily smokers. The proportion of e-cigarette users was 2.1 % (312/14530).

**Table 1:** The characteristics of participants in this study.

Characteristics of participants	
Number	14530
Age, year (mean ± SD)	51.1 ± 16.5
Gender, female (n, %)	7995 (55.0 %)
Response for self-report (n, %)	14454 (99.5 %)
Non-smoker, self-report (n, %)	11601 (80.3 %)
SR– (n, %)	9629 (83.0 %)
Non-smoker with unclear response (n, %)	3 (0.0 %)
SR+ (n, %)	1969 (17.0 %)
Current smoker, self-report (n, %)	2853 (19.7 %)
Daily smoker, self-report (n, %)	2484 (87.1 %)
Non-daily smoker, self-report (n, %)	369 (12.9 %)
Usage of E–cigarette (n, %)	312 (2.1 %)

SR–, non-SHS exposure participants; SR+, SHS exposure participants; SHS, second-hand smoke.

### 3.2 The results of urinary cotinine concentrations

Table 2 displays the results of UCs according to self-reported smoking status and self-reported SHS exposure. The median UCs of SR–, SR+, daily smokers, and non-daily smokers were 0.6 μg/L (the first and third quartiles, 0.4 μg/L–1.1 μg/L), 1.2 μg/L (0.6 μg/L–2.8 μg/L), 1325.7 μg/L (805.0 μg/L–1860.0 μg/L), and 355.0 μg/L (95.4 μg/L–908.0 μg/L), respectively. Among them, participants with <0.3 μg/L of UC were 16.7 % (1608/9629), 5.4 % (106/1969), 0.0 % (0/2484), and 0.3 % (1/369), respectively.

**Table 2:** The urinary cotinine concentration and depression prevalence using PHQ-9 scores according to the self-reported smoking status and SHS exposure.

Subgroup	Urinary cotinine [median (Q1–Q3), μg/L]	≤0.3 μg/L (n, %)	PHQ-9 score	the depression prevalence^a^	*p* value^b^
SR–	0.6 (0.4–1.1)	1608 (16.7 %)	1 (0–3)	520 (5.4 %)	
(n = 9629)					
SR+	1.2 (0.6–2.8)	106 (5.4 %)	2 (0–4)	106 (5.4 %)	0.9994.
(n = 1969)					
Total non-smokers^c^	0.7 (0.4–1.3)	1714 (14.8 %)	1 (0–4)	626 (5.4 %)	
(n = 11601)					
Daily smokers	1325.7 (805.0–1860.0)	0 (0.0 %)	1 (0–4)	194 (7.8 %)	<0.001
(n = 2484)					
Non-daily smokers	355.0 (95.4–908.0)	1 (0.3 %)	1 (0–4)	22 (6.0 %)	0.6400
(n = 369)					
Total current smokers	1230.0 (668.4–1778.3)	1 (0.0 %)	1 (0–4)	216 (7.6 %)	
(n = 2853)					
Overall^c^	0.9 (0.4–6.1)	1722 (11.9 %)	1 (0–3)	851 (5.8 %)	
(n = 14531)					

*Note:*^a^ The depression prevalence was estimated as the percentage of participants with a PHQ-9 score ≤10 out of the total participants in each subgroup. ^b^ Compared with the depression prevalence in SR–, each *p* value was calculated using Chi-square test. ^C^ Total non-smokers and overall participants included 3 participants with unclear responses.

Q1–Q3, first and third quartiles; SR–, non-SHS exposure; SR+, SHS exposure; SHS, second-hand smoke.

### 3.3 The results of the depression prevalence using the PHQ-9 score

Table 2 displays the depression prevalence using the PHQ-9 score according to self-reported smoking status and self-reported SHS exposure. The depression prevalence among SR–, SR+, daily smokers, and non-daily smokers was 5.4 % (520/9629), 5.4 % (106/1969), 7.8 % (194/2484), and 6.0 % (22/369), respectively. Compared to SR–, daily smokers showed a higher depression prevalence (7.8 % vs. 5.4 %, *p* < 0.001), whereas SR+ and non-daily smokers showed a very small and non-significant difference.

Subgroup Analysis for the Depression Prevalence According to Gender, Self-reported SHS Exposure, and Categorized Urinary Cotinine Concentrations among Non-smokers.

**Fig. 2:**
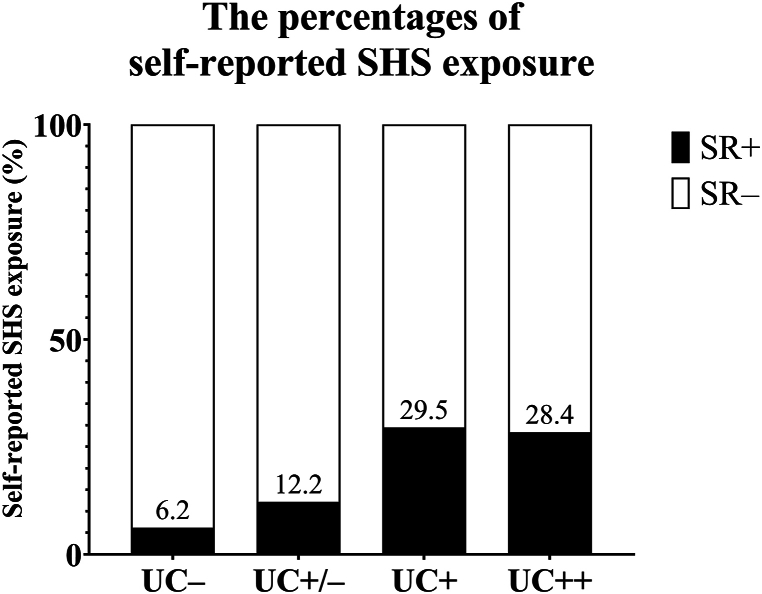
The percentages of self-reported SHS exposure according to categorized UCs among non-smokers. UC, urinary cotinine concentration; SR–, non-SHS exposure participants; SR+, SHS exposure participants; SHS, second-hand smoke.

Table 3 displays the results of subgroup analysis for the depression prevalence according to gender, self-reported SHS exposure, and categorized UCs among non-smokers. The percentages of SR+ in the UC–, UC±, UC+, and UC++ subgroups were 6.2 % (106/1714), 12.2 % (732/6024), 29.5 % (948/3217), and 28.4 % (183/644), respectively (Fig. 2).

**Table 3:** Subgroup analysis for the depression prevalence among non-smokers.

Urinary cotinine	Gender	SR– (n, %)	Depression^a^ (n,%)	*p* value^b^	SR+ (n, %)	Depression (n,%)	*p* value^b^	Total (n, %)	Depression (n,%)	*p* value^b^
UC– (≤0.3 μg/L)	Female (%)	1200 (70.0 %)	88 (7.3 %)		62 (3.6 %)	2 (3.2 %)		1262 (73.6 %)	90 (7.1 %)	
	Male (%)	408 (23.8 %)	16 (3.9 %)^c^		44 (2.6 %)	1 (2.3 %)		452 (26.4 %)b	17 (3.8 %)	
	Total (%)	1608 (93.8 %)	104 (6.5 %)		106 (6.2 %)	3 (2.8 %)		1714 (100.0 %)	107 (6.2 %)	
UC± (0.4 μg/L–0.9 μg/L)	Female (%)	3453 (57.3 %)	206 (6.0 %)	0.098	409 (6.8 %)	26 (6.4 %)	0.562	3862 (64.1 %)	232 (6.0 %)	0.161
	Male (%)	1838 (30.5 %)	44 (2.4 %)^c^	0.090	323 (5.4 %)	10 (3.1 %)	1.000	2162 (35.9 %)	54 (2.5 %)^c^	0.151
	Total (%)	5291 (87.8 %)	250 (5.0 %)	0.007	732 (12.2 %)	36 (4.9 %)	0.462	6024 (100.0 %)	286 (4.7 %)	0.015
UC+ (1.0 μg/L–11.9 μg/L)	Female (%)	1468 (45.6 %)	102 (6.9 %)	0.706	564 (17.5 %)	39 (6.9 %)	0.415	2034 (63.2 %)	141 (6.9 %)	0.113
	Male (%)	801 (24.9 %)	24 (3.0 %)^c^	0.399	384 (11.9 %)	12 (3.1 %)^c^	1.000	1185 (36.8 %)	36 (3.0 %)^c^	0.439
	Total (%)	2269 (70.5 %)	126 (5.6 %)	0.241	948 (29.5 %)	51 (4.5 %)	0.354	3217 (100.0 %)	177 (5.5 %)	0.305
UC++ (≥12.0 μg/L)	Female (%)	248 (38.5 %)	32 (12.9 %)	0.005	105 (16.3 %)	14 (13.3 %)	0.054	353 (54.8 %)	46 (13.0 %)	0.447
	Male (%)	213 (33.1 %)	8 (3.8 %)^c^	1.000	78 (12.1 %)	2 (2.6 %)^c^	1.000	291 (45.2 %)	10 (3.4 %)^c^	1.000
	Total (%)	461 (71.6 %)	40 (8.7 %)	0.119	183 (28.4 %)	16 (8.7 %)	0.082	644 (100.0 %)	56 (8.7 %)	0.045

*Note:*^a^ The depression prevalence was estimated as the percentage of participants with a PHQ-9 score ≤10 out of the total participants in each subgroup. ^b^ Compared with the depression prevalence in the UC– in the same SR and gender, each *p* value was calculated using Fisher's exact test.

^C^ Compared with the depression prevalence among females in the same UC category and SR, each *p* value was significant. ^d^.

UC, urinary cotinine concentration; SR–, non-SHS exposure participants; SR+, SHS exposure participants; SHS, second-hand smoke.

When UC– was used as the reference subgroup, the UC++ subgroup showed a higher depression prevalence (8.7 % [56/644] vs. 6.2 % [107/1714], *p* = 0.045), whereas the UC ± subgroup showed a lower prevalence (4.7 % [286/6024] vs. 6.2 % [107/1714], *p* = 0.015). The depression prevalence in the UC + subgroup was comparable to that in the UC– subgroup (5.5 % [177/3217] vs. 6.2 % [107/1714], *p* > 0.10).

**Fig. 3:**
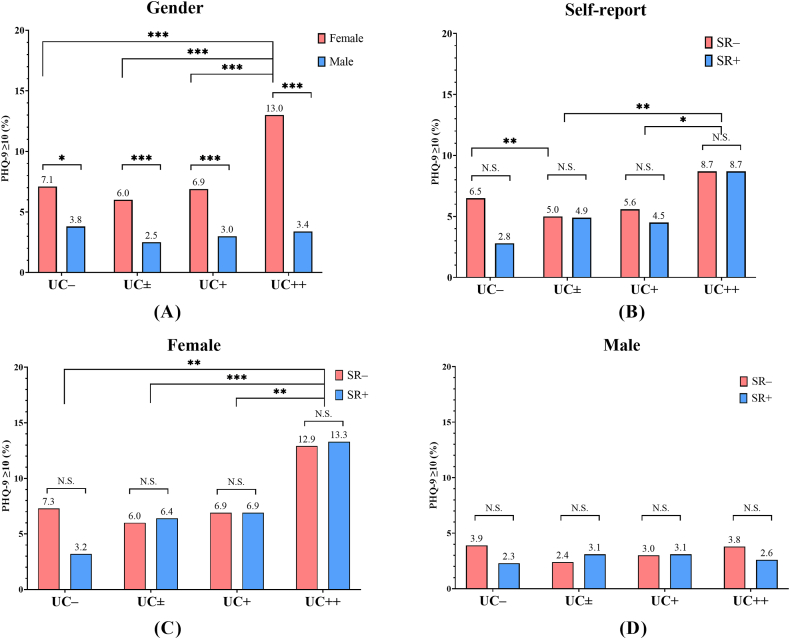
The comparison of the depression prevalence according to sex (A), self-reported SHS exposure (B), and categorized UCs among non-smokers. According to sex, the depression prevalence among females (C) and males (D) are shown. * = *p* value < 0.05; ** = *p* value < 0.01; *** = *p* value < 0.001, respectively. N.S., not significant; UC, urinary cotinine concentration; SR–, non-SHS exposure participants; SR+, SHS exposure participants; SHS, second-hand smoke.

In the same UC categories, the depression prevalence was higher among females than among males (Fig. 3A); however, there was no difference between SR+ and SR– (Fig. 3B). Females in the UC++ subgroup showed a higher depression prevalence than females in the UC– subgroup (13.0 % [46/353] vs. 7.1 % [90/1262], *p* < 0.001) (Fig. 3C), whereas females in the UC ± subgroup (6.0 % [232/3862]) and UC + subgroup (6.9 % [141/2034]) did not show a difference (Fig. 3C). In the current study, the depression prevalence among males was comparable irrespective of the UC category (UC– vs. UC ± vs. UC + vs. UC++, 3.8 % [17/452] vs. 2.5 % [54/2162] vs. 3.0 % [36/1185] vs. 3.4 % [10/291], *p* > 0.10) (Fig. 3D).

Subgroup Analysis for the Severe Stress Rate According to Gender, Self-reported SHS Exposure, and Categorized Urinary Cotinine Concentrations among Non-smokers.

**Fig. 4:**
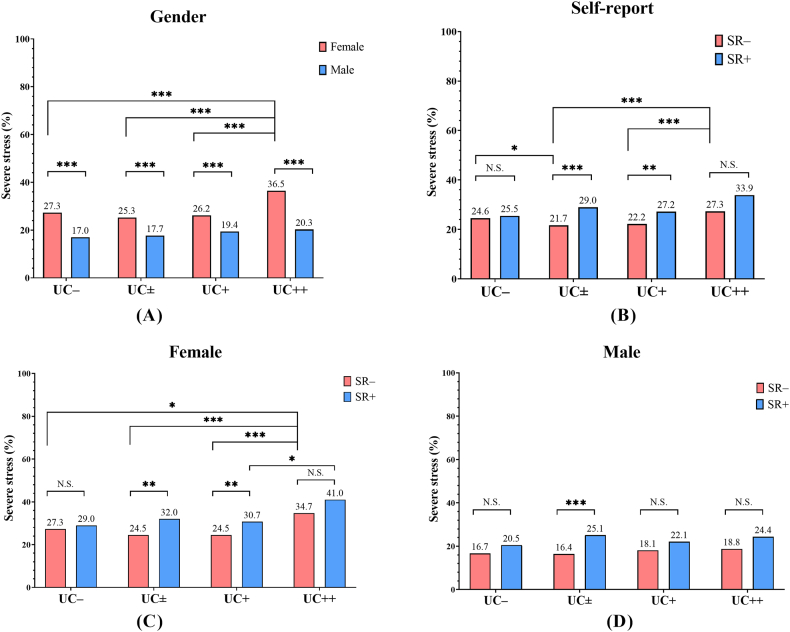
The comparison of the severe stress rate according to sex (A), self-reported SHS exposure (B), and categorized UCs among non-smokers. According to sex, the severe stress rate among females (C) and males (D) are shown. * = *p* value < 0.05; ** = *p* value < 0.01; *** = *p* value < 0.001, respectively. N.S., not significant; UC, urinary cotinine concentration; SR–, non-SHS exposure participants; SR+, SHS exposure participants; SHS, second-hand smoke.

Table 4 and Fig. 4 describe the results of subgroup analysis for the severe stress rate according to gender, self-reported SHS exposure, and categorized UCs among non-smokers. When UC– was used as the reference subgroup, the UC++ subgroup showed a higher severe stress rate (29.2 % [188/644] vs. 24.6 % [422/1714], *p* = 0.031), whereas the UC± (22.6 % [1359/6024] vs. 24.6 % [422/1714], *p* = 0.059) and UC + subgroups (23.7 % [762/3219] vs. 24.6 % [422/1714], *p* > 0.10) did not show a significantly different rate.

**Table 4:** Subgroup analysis for the severe stress rate according to sex, self-reported SHS exposure, and categorized UCs among non-smokers.

Urinary cotinine	Gender	SR– (n, %)	Severe stress (n,%)	*p* value^a^	SR+ (n, %)	Severe stress (n,%)	*p* value^a^	Total (n, %)	Severe stress (n,%)	*p* value^a^
UC– (≤0.3 μg/L)	Female (%)	1200 (70.0 %)	327 (27.3 %)		62 (3.6 %)	18 (29.0 %)		1262 (73.6 %)	345 (27.3 %)	
	Male (%)	408 (23.8 %)	68 (16.7 %)^b^		44 (2.6 %)	9 (20.5 %)		452 (26.4 %)	77 (17.0 %)^b^	
	Total (%)	1608 (93.8 %)	395(24.6 %)		106 (6.2 %)	27 (25.5 %)		1714 (100.0 %)	422 (24.6 %)	
UC± (0.4 μg/L–0.9 μg/L)	Female (%)	3453 (57.3 %)	845 (24.5 %)	0.057	409 (6.8 %)	131(32.0 %)c	0.770	3862 (64.1 %)	976 (25.3 %)	0.148
	Male (%)	1838 (30.5 %)	302 (16.4 %)^b^	0.941	323 (5.4 %)	81 (25.1 %)^b,c^	0.579	2162 (35.9 %)	383 (17.7 %)^b^	0.786
	Total (%)	5291 (87.8 %)	1147 (21.7 %)	0.017	732 (12.2 %)	212 (29.0 %)^c^	0.492	6024 (100.0 %)	1359 (22.6 %)	0.079
UC+ (1.0 μg/L–11.9 μg/L)	Female (%)	1468 (45.6 %)	359 (24.5 %)	0.101	564 (17.5 %)	173 (30.7 %)^c^	0.885	2034 (63.2 %)	532 (26.2 %)	0.466
	Male (%)	801 (24.9 %)	145 (18.1 %)^b^	0.577	384 (11.9 %)	85 (22.1 %)^b^	1.000	1185 (36.8 %)	230 (19.4 %)	0.289
	Total (%)	2269 (70.5 %)	504 (22.2 %)	0.090	948 (29.5 %)	258 (27.2 %)^c^	0.818	3219 (100.0 %)	762 (23.7 %)	0.462
UC++ (≥12.0 μg/L)	Female (%)	248 (38.5 %)	86 (34.7 %)	0.021	105 (16.3 %)	43 (41.0 %)	0.137	353 (54.8 %)	129 (36.5 %)	<0.001
	Male (%)	213 (33.1 %)	40 (18.8 %)^b^	0.508	78 (12.1 %)	19 (24.4 %)^b^	0.661	291 (45.2 %)^b^	59 (20.3 %)	0.286
	Total (%)	461 (71.6 %)	126 (27.3 %)	0.248	183 (28.4 %)	62 (33.9 %)	0.148	644 (100.0 %)	188 (29.2 %)	0.027

Notes: ^a^ Compared with the severe stress rate among UC– in the same SR and sex, each *p* value was calculated using Fisher's exact test. ^b^ Compared with the severe stress rate among females in the same UC category and SR, each *p* value was significant. ^C^ Compared with the severe stress rate among SR– in the same UC category and sex, each *p* value was significant.

UC, urinary cotinine concentration; SR–, non-SHS exposure participants; SR+, SHS exposure participants; SHS, second-hand smoke.

In the same UC categories, the rates of severe stress were higher among females than among males (Fig. 4A). Furthermore, the rates of severe stress were higher among SR + compared to SR– in the UC± and UC + subgroups (SR+/UC ± vs. SR–/UC±, 29.0 % [212/732] vs. 21.7 % [1147/5291], *p* < 0.001; SR+/UC + vs. SR–/UC+, 27.2 % [258/948] vs. 22.2 % [504/2269], *p* = 0.003) (Fig. 4B). However, there was no significant difference in UC– and UC++ subgroup (SR+/UC– vs. SR–/UC–, 25.5 % [27/106] vs. 24.6 % [395/1608], *p* > 0.10; SR+/UC++ vs. SR–/UC++, 33.9 % [62/183] vs. 27.3 % [126/461], *p* = 0.054) (Fig. 4B). Females in the UC±, UC+, and UC++ subgroups showed higher rates of severe stress than females in the UC– subgroup (Fig. 4C), whereas males were comparable irrespective of the UC category (Fig. 4D).

### 3.4 Binominal logistic regression analysis

Table 5 displays the results of binominal logistic regression analysis for the depression prevalence or severe stress rate with age, gender, education level, household income, type of house, type of marriage, SR, categorical UC, and quantitative UC, as covariates. Gender, education status, household income, and type of marriage showed the independent association with the depression prevalence. Age, gender, household income, as well as SR showed the independent association with the severe stress rate. However, neither categorized UC nor quantitative UC showed the independent association with the depression prevalence or severe stress rate.

**Table 5:** Binominal logistic regression analysis for the depression prevalence or severe stress rate.

Variables	The depression prevalence				The severe stress rate			
	B	S.E	*p* value	OR	B	S.E	*p* value	OR
Age	−0.005	0.004	0.232	0.995	−0.024	0.002	<0.001	0.976
Gender	0.860	0.108	<0.001	2.363	0.453	0.05	<0.001	1.573
Education level	−0.231	0.053	<0.001	0.793	−0.033	0.028	0.244	0.968
Household income	−0.447	0.047	<0.001	0.639	−0.127	0.024	<0.001	0.881
Type of house	−0.062	0.053	0.240	0.940	0.003	0.030	0.931	1.003
Type of marriage	0.031	0.014	0.030	1.032	0.017	0.013	0.209	1.017
SR+	0.109	0.078	0.164	1.115	0.152	0.041	<0.001	1.164
UC, categorical	−0.010	0.020	0.615	0.990	0.006	0.011	0.577	1.006
UC, quantitative	0.000	0.000	0.209	1.000	0.000	0.000	0.446	1.000

UC, urinary cotinine concentration; SR+, SHS exposure participants; S.E, systemic error; OR, odds ratio.

## 4 Discussion

Several hypotheses based on two different approaches have been proposed to explain the relationship between depression and SHS exposure. One is the hormonal and neuronal-based hypothesis, and the other is the emotional stress-based hypothesis. Although these hypotheses are not mutually exclusive, the dose-response relationship between SHS exposure biomarkers and the depression prevalence or stress requires an in-depth examination to establish appropriate models between Depression and SHS exposure.

This is the first study to show a paradoxical reduction in the depression prevalence and severe stress rate in the minimal-dose SHS-exposed subgroup compared to the non–SHS–exposed group among non-smokers without self-reported SHS exposure. In this study, the UC ± subgroup showed a lower depression prevalence (Fig. 3B) and a lower severe stress rate (Fig. 4B) than the UC– subgroup. Furthermore, the dose-response relationship between the SHS exposure biomarker and the depression prevalence was not linear. These results are in contrast to previous studies, which reported a linear relationship between self-reported SHS exposure frequency and the depression prevalence [13,33,34].

This discrepancy may be explained by the difference between self-reported SHS exposure and objectively measured UC. Compared to the measured UC-based classification, the self-report-based classification significantly underestimated non-smokers with SHS exposure. The percentage of non-smokers with self-reported SHS exposure in the UC ± subgroup was markedly lower than those in the UC+ and UC++ subgroups (Fig. 2). Furthermore, among the same UC ± category, the SR + subgroup showed a higher severe stress rate compared with the SR– subgroup in the current study (Fig. 4). Therefore, the SR+/UC ± subgroup, which had a higher severe stress rate, might be overrepresented among the UC ± subgroup in the self-report-based studies compared to the measured biomarker-based studies.

Although, statistical significance was not satisfied among all UC categories, the SR + subgroup showed a higher severe stress rate compared with the SR– subgroup of the same UC category in the current study (Fig. 4). Psychological and physiological stress responses vary widely among individuals [35]. If the SR– subgroup was considered as the subgroup with relative tolerance for SHS exposure-related stress and the SR + subgroup was considered as the susceptible subgroup for SHS exposure-related stress, it could be explained by a paradoxical reduction in the depression prevalence and severe stress rate in the UC ± subgroup compared to the UC– subgroup. Because the SR–/UC– subgroup had no or minimal opportunity to respond to SHS exposure, the SR + subgroup could not be clearly excluded. Thus, the SR–/UC ± subgroup, which consisted of responded SR– participants, could have a lower depression prevalence and severe stress rate than non-smokers without SHS exposure (Fig. 3, Fig. 4B).

The relationship between gender and exposure to a stressor has been studied [[36], [37], [38], [39]], and individual differences in psychological responses to stress have been shown to be potentially significant risk factors for depression. In the current study, females were more susceptible to SHS exposure-related stress than males. However, an increase in the severe stress rate was observed only in the UC++ subgroup among female non-smokers (Fig. 4C). In contrast, there was no significant increase in the severe stress rate among male non-smokers (Fig. 4D). These results suggest that females are more susceptible to SHS exposure-related stress than males.

Interestingly, the UC++ subgroup, which was indicated as high-dose exposure similar to the current smoker level, showed a higher depression prevalence than other subgroups, and these results were consistent with a previous German study [40]. This phenomenon can be explained by the widely accepted stress-disease model, the wear and tear model [41,42]. According to the model, increased emotional responsiveness to daily stressors increases the overall emotional distress and the likelihood of developing depression [41].

On the other hand, there were some obstacles in explaining the relationship between the measured UC and depression prevalence and severe stress rate using hormonal and neuronal-based hypotheses. Marked gender difference, a paradoxical reduction in the UC ± subgroup, and non-linear dose-response relationship between the measured UC and SHS exposure are difficult to explain via hormonal and neuronal-based hypotheses. Therefore, our study indicates that the emotional stress-based model is more appropriate for explaining the relationship between depression and SHS exposure.

The current study has some limitations. First, this was a cross-sectional, observational study. Therefore, the cause-and-effect relationship between stress responses and SHS exposure could not be determined. Second, this study used only UC as a tobacco-specific biomarker. We observed about 5 % of SR– participants were belonged to the UC++ group, estimated to be daily or intermittent smokers. Serum cotinine is a widely used biomarker for smoking status verification [43], and urinary 4-(Methylnitrosamino)-1-(3-pyridyl)-1-butanol (NNAL) is more sensitive than UC for detecting SHS exposure over a much longer period [43]. Thus, the concordance between self-reported SHS exposure and measured biomarker concentrations might change according to tobacco-specific biomarkers [44]. Third, the results cannot be generalized because only adult Koreans were included in the current study. The sample size was not enough to identify the statistical significance of subgroup analysis, although the SR + subgroup showed a higher severe stress rate compared with the SR– subgroup at the same biological level of SHS exposure. However, further studies are required to address these limitations.

## 5 Conclusion

In conclusion, the current study showed that the SR + subgroup had a higher severe stress rate than the SR– subgroup in the same UC category. Additionally, this study showed that females were more susceptible to SHS exposure-related stress than males. Furthermore, the study showed a paradoxical reduction in the depression prevalence and severe stress rate in the minimal-dose SHS-exposed subgroup compared to the non–SHS–exposed group among non-smokers without self-reported SHS exposure. Therefore, this study suggests that an emotional stress-based model is more appropriate for explaining the relationship between depression and SHS exposure.

## Ethics approval and consent to participate

The institutional review board of Wonkwang University Hospital approved this study (IRB file no. 2022–03–023). The need for informed consent was waived, because the project was aimed to perform a comparative analysis using a public database.

## Consent for publication

Not applicable.

## Data availability statement

The datasets used in the current study were available in the Korean National Health and Nutrition Examination Survey (KNHANES) conducted in 2014, 2016, and 2018 (https://knhanes.kdca.go.kr/knhanes/eng/index.do). The dataset recruitment process is shown in the Supplementary Table S1.

## Research funding

This study was funded by 10.13039/501100002569Wonkwang University in 2023.

## CRediT authorship contribution statement

**Hyun-Seung Lee:** Writing – review & editing, Writing – original draft, Validation, Project administration, Methodology, Investigation, Funding acquisition, Formal analysis, Data curation. **Young-Jin Lee:** Writing – review & editing. **Ji-Hyun Cho:** Writing – review & editing. **Do-Sim Park:** Writing – review & editing, Validation, Supervision.

## Declaration of competing interest

The authors declare that they have no known competing financial interests or personal relationships that could have appeared to influence the work reported in this paper.

## List of abbreviations

GC-MS/MS: as chromatography-tandem mass spectrometry
HPLC-MS/MS: high-performance liquid chromatography-tandem mass spectrometry
KNHANES: Korean National Health and Nutrition Examination Survey
LoD: the limit of detection
NNAL: 4-(Methylnitrosamino)-1-(3-pyridyl)-1-butanol
PHQ-9: Patient Health Questionnaire-9
SHS: second-hand smoke
SR+: SHS exposure participants
SR–: non-SHS exposure participants
UC: urinary cotinine concentration

## References

[1] Polosa R., Thomson N.C. (2013). Smoking and asthma: dangerous liaisons. Eur. Respir. J..

[2] Ebbert J.O., Croghan I.T., Schroeder D.R., Murawski J., Hurt R.D. (2007). Association between respiratory tract diseases and second-hand smoke exposure among never smoking flight attendants: a cross-sectional survey. Environ. Health.

[3] Gandini S., Botteri E., Iodice S., Boniol M., Lowenfels A.B., Maisonneuve P. (2008). Tobacco smoking and cancer: a meta-analysis. Int. J. Cancer.

[4] Taylor G.M., Munafò M.R. (2019). Does smoking cause poor mental health?. Lancet Psychiatr..

[5] Wootton R.E., Richmond R.C., Stuijfzand B.G., Lawn R.B., Sallis H.M., Taylor G.M. (2020). Evidence for causal effects of lifetime smoking on risk for depression and schizophrenia: a Mendelian randomization study. Psychol. Med..

[6] Weinberger A.H., Kashan R.S., Shpigel D.M., Esan H., Taha F., Lee C.J. (2017). Depression and cigarette smoking behavior: a critical review of population-based studies. Am. J. Drug Alcohol Abuse.

[7] Petty F. (1995). GABA and mood disorders: a brief review and hypothesis. J. Affect. Disord..

[8] Bandiera F.C., Arheart K.L., Caban-Martinez A.J., Fleming L.E., McCollister K., Dietz N.A. (2010). Second-hand smoke exposure and depressive symptoms. Psychosom. Med..

[9] Nunes S.O., Vargas H.O., Brum J., Prado E., Vargas M.M., Castro M.R. (2011). A comparison of inflammatory markers in depressed and nondepressed smokers. Nicotine Tob. Res..

[10] Edwards A.C., Kendler K.S. (2012). A twin study of depression and nicotine dependence: shared liability or causal relationship?. J. Affect. Disord..

[11] Fu Q., Heath A.C., Bucholz K.K., Lyons M.J., Tsuang M.T., True W.R. (2007). Common genetic risk of major depression and nicotine dependence: the contribution of antisocial traits in a United States veteran male twin cohort. Twin Res. Hum. Genet..

[12] National Center for Chronic Disease Prevention and Health Promotion Office on Smoking and Health (2014).

[13] Noguchi T., Nakagawa-Senda H., Tamai Y., Nishiyama T., Watanabe M., Hosono A. (2020). Association between second-hand smoke exposure and depressive symptoms among Japanese adults: a cross-sectional study. J. Epidemiol..

[14] Han C., Liu Y., Gong X., Ye X., Zhou J. (2019). Relationship between secondhand smoke exposure and depressive symptoms: a systematic review and dose-response meta-analysis. Int. J. Environ. Res. Publ. Health.

[15] Zeng Y.N., Li Y.M. (2016). Second-hand smoke exposure and mental health in adults: a meta-analysis of cross-sectional studies. Soc. Psychiatr. Psychiatr. Epidemiol..

[16] Patten S.B., Williams J.V., Lavorato D.H., Woolf B., Wang J.L., Bulloch A.G. (2018). Major depression and second-hand smoke exposure. J. Affect. Disord..

[17] Jung S.J., Shin A., Kang D. (2015). Active smoking and exposure to second-hand smoke and their relationship to depressive symptoms in the Korea national health and nutrition examination survey (KNHANES). BMC Publ. Health.

[18] Nakata A., Takahashi M., Ikeda T., Hojou M., Nigam J.A., Swanson N.G. (2008). Active and passive smoking and depression among Japanese workers. Prev. Med..

[19] Arheart K.L., Lee D.J., Fleming L.E., LeBlanc W.G., Dietz N.A., McCollister K.E. (2008). Accuracy of self-reported smoking and second-hand smoke exposure in the US workforce: the National Health and Nutrition Examination Surveys. J. Occup. Environ. Med..

[20] Lee H.S., Cho J.H., Lee Y.J., Park D.S. (2022). Effect of second-hand smoke exposure on establishing urinary cotinine-based optimal cut-off values for smoking status classification in Korean adults. Int. J. Environ. Res. Publ. Health.

[21] Bandiera F.C., Arheart K.L., Caban-Martinez A.J., Fleming L.E., McCollister K., Dietz N.A. (2010). Second-hand smoke exposure and depressive symptoms. Psychosom. Med..

[22] Hamer M., Stamatakis E., Batty G.D. (2010). Objectively assessed second-hand smoke exposure and mental health in adults: cross-sectional and prospective evidence from the Scottish Health Survey. Arch. Gen. Psychiatr..

[23] Bot M., Vink J.M., Willemsen G., Smit J.H., Neuteboom J., Kluft C. (2013). Exposure to second-hand smoke and depression and anxiety: a report from two studies in The Netherlands. J. Psychosom. Res..

[24] Park M.B., Kwan Y., Sim B., Lee J. (2021). Association between urine cotinine and depressive symptoms in non-smokers: national representative sample in Korea. J. Affect. Disord..

[25] Okekunle A.P., Asowata J.O., Lee J.E., Akpa O.M. (2021). Association of Environmental tobacco smoke exposure with depression among non-smoking adults. BMC Publ. Health.

[26] Kim S.J., Han K.T., Lee S.Y., Chun S.Y., Park E.C. (2015). Is secondhand smoke associated with stress in smokers and non-smokers?. BMC Publ. Health.

[27] Asbridge M., Ralph K., Stewart S. (2013). Private space second-hand smoke exposure and the mental health of non-smokers: a cross-sectional analysis of Canadian adults. Addict. Behav..

[28] Michal M., Wiltink J., Reiner I., Kirschner Y., Wild P.S., Schulz A. (2013). Association of mental distress with smoking status in the community: results from the Gutenberg Health Study. J. Affect. Disord..

[29] Bandiera F.C. (2011). What are candidate biobehavioral mechanisms underlying the association between second-hand smoke exposure and mental health?. Med. Hypotheses.

[30] Jefferis B.J., Lowe G.D., Welsh P., Rumley A., Lawlor D.A., Ebrahim S. (2010). Second-hand smoke (SHS) exposure is associated with circulating markers of inflammation and endothelial function in adult men and women. Atherosclerosis.

[31] Berk M., Williams L.J., Jacka F.N., O'Neil A., Pasco J.A., Moylan S. (2013). So depression is an inflammatory disease, but where does the inflammation come from?. BMC Med..

[32] Kroenke K., Spitzer R.L., Williams J.B. (2001). The PHQ-9: validity of a brief depression severity measure. J. Gen. Intern. Med..

[33] Ye X., Li L., Gao Y., Zhou S., Yang Y., Chen S. (2015). Dose-response relations between second-hand smoke exposure and depressive symptoms among middle-aged women. Psychiatr. Res..

[34] Huang J., Xu B., Guo D., Jiang T., Huang W., Liu G. (2018). Dose-response relationships between second-hand smoke exposure and depressive symptoms among adolescents in guangzhou, China. Int. J. Environ. Res. Publ. Health.

[35] Crosswell A.D., Lockwood K.G. (2020). Best practices for stress measurement: how to measure psychological stress in health research. Health Psychol. Open.

[36] Suzuki A., Akamatsu R. (2014). Sex differences in relationship between stress responses and lifestyle in Japanese workers. Saf Health Work.

[37] Neufeld R.W., Davidson P.O. (1974). Sex differences in stress response: a multivariate analysis. J. Abnorm. Psychol..

[38] Verma R., Balhara Y.P., Gupta C.S. (2011). Gender differences in stress response: role of developmental and biological determinants. Ind. Psychiatr. J..

[39] Kajantie E., Phillips D.I. (2006). The effects of sex and hormonal status on the physiological response to acute psychosocial stress. Psychoneuroendocrinology.

[40] Erdsiek F., Brzoska P. (2020). Is exposure to secondhand smoke associated with current depression (PHQ-8) among never-smokers? Results from a survey among German adults. BMC Publ. Health.

[41] Charles S.T., Piazza J.R., Mogle J., Sliwinski M.J., Almeida D.M. (2013). The wear and tear of daily stressors on mental health. Psychol. Sci..

[42] McEwen B.S. (1998). Stress, adaptation, and disease. Allostasis and allostatic load. Ann. N. Y. Acad. Sci..

[43] Benowitz N.L., Bernert J.T., Foulds J., Hecht S.S., Jacob P., Jarvis M.J. (2020). Biochemical verification of tobacco use and abstinence: 2019 update. Nicotine Tob. Res..

[44] Lee H.S. (2022). Diagnostic performance evaluation of the novel index combining urinary cotinine and 4-(Methylnitrosamino)-1-(3-pyridyl)-1-butanol in smoking status verification and usefulness for trend monitoring of tobacco smoking exposure. Int. J. Environ. Res. Publ. Health.

