# Supplementary material for: Analysis of patient health questionnaire-9 (PHQ-9) based depression prevalence according to a discordance between quantitative urinary cotinine levels and self-report of second-hand smoke exposure among adults: A cross-sectional study
Source: Heliyon. 2024 May 29;10(11):e32125. doi: 10.1016/j.heliyon.2024.e32125 (PMC11176832; doi:10.1016/j.heliyon.2024.e32125)
Supplement: Multimedia component 1 [file mmc1.pdf]

Supplemental Table 1. Dataset recruitment

Dataset	Survey project	Year	Survey Institution
2014 KNHANES	The Sixth Korea National Health and Nutrition Examination Survey (KNHANES VI-2)	2014	Korea Centers for Disease Control and Prevention
2016 KNHANES	The Sixth Korea National Health and Nutrition Examination Survey (KNHANES VII-1)	2016	Korea Centers for Disease Control and Prevention
2018 KNHANES	The Seventh Korea National Health and Nutrition Examination Survey (KNHANES VII-3)	2018	Korea Centers for Disease Control and Prevention

Access link https://knhanes.kdca.go.kr/knhanes/eng/index.do
