# Supplementary material for: Analysis of patient health questionnaire-9 (PHQ-9) based depression prevalence according to a discordance between quantitative urinary cotinine levels and self-report of second-hand smoke exposure among adults: A cross-sectional study
Source: Heliyon. 2024 May 29;10(11):e32125. doi: 10.1016/j.heliyon.2024.e32125 (PMC11176832; doi:10.1016/j.heliyon.2024.e32125)
Supplement: Multimedia component 2 [file mmc2.pdf]

Supplemental Table 2. PHQ-9 and severe stress related questionnaire and variables

Over the last 2 weeks, how often have you been botheredby any of the following problems?

Questionnaire	Variables	Not at all	Several days	More than half the days	Nearly every day
Little interest or pleasure in doing things	BP_PHQ_1	0	1	2	3
Feeling down, depressed, or hopeless	BP_PHQ_2	0	1	2	3
Trouble falling or staying asleep, or sleeping too much	BP_PHQ_3	0	1	2	3
Feeling tired or having little energy	BP_PHQ_4	0	1	2	3
Poor appetite or overeating	BP_PHQ_5	0	1	2	3
Feeling bad about yourself or that you are a failure or have let yourself or your family down	BP_PHQ_6	0	1	2	3
Trouble concentrating on things, such as reading the newspaper or watching television	BP_PHQ_7	0	1	2	3
Moving or speaking so slowly that other people could have noticed? Or the opposite being so fidgety or restlessthat you have been moving around a lot more than usual	BP_PHQ_8	0	1	2	3
Thoughts that you would be better off dead or of hurting yourself in some way	BP_PHQ_9	0	1	2	3
PHQ-9 score	mh_PHQ_S	Sum of BP_PHQ variables			

How much stress do you feel in your daily life?

Questionnaire	Variables	Not at all	Mild	Severe	Very severe
Daily life stress	BP1	4	3	2	1
Severe stress	BP1	1 or 2 of BP1 score			
