# Supplementary material for: Analysis of patient health questionnaire-9 (PHQ-9) based depression prevalence according to a discordance between quantitative urinary cotinine levels and self-report of second-hand smoke exposure among adults: A cross-sectional study
Source: Heliyon. 2024 May 29;10(11):e32125. doi: 10.1016/j.heliyon.2024.e32125 (PMC11176832; doi:10.1016/j.heliyon.2024.e32125)
Supplement: Multimedia component 3 [file mmc3.pdf]

Supplemental Table 3. Social status related questionnaire and variables

Questionnaire	Variables	1	2	3	4
Education	Edu	Primary school	Middle school	High school	College or higher
House income	ho_incom	Fourth quartile	Third quartile	Second quratile	First qurtile
Type of residence	ive_t	Single unit	Apartment	Multi unit	Others
Type of marriage	marri_2	Married, unseparated	Married, separated	Bereaved	Divorced

9
Unknown
